# Age-Dependent Asymmetry of Wrist Position Sense Is Not Influenced by Stochastic Tactile Stimulation

**DOI:** 10.3389/fnhum.2020.00065

**Published:** 2020-03-03

**Authors:** Anna-Maria Georgarakis, Harshal A. Sonar, Mike D. Rinderknecht, Werner L. Popp, Jaime E. Duarte, Olivier Lambercy, Jamie Paik, Bernard J. Martin, Robert Riener, Verena Klamroth-Marganska

**Affiliations:** ^1^Sensory-Motor Systems (SMS) Lab, Department of Health Sciences and Technology (D-HEST), Institute of Robotics and Intelligent Systems (IRIS), ETH Zurich, Zurich, Switzerland; ^2^Reharobotics Group, Medical Faculty, Spinal Cord Injury Center, Balgrist University Hospital, University of Zurich, Zurich, Switzerland; ^3^Reconfigurable Robotics Laboratory (RRL), Institute of Mechanical Engineering, École Polytechnique Fédérale de Lausanne, Lausanne, Switzerland; ^4^Rehabilitation Engineering Laboratory (RELab), Department of Health Sciences and Technology (D-HEST), Institute of Robotics and Intelligent Systems (IRIS), ETH Zurich, Zurich, Switzerland; ^5^Department of Industrial and Operations Engineering (IOE), Center for Ergonomics, University of Michigan, Ann Arbor, MI, United States; ^6^School of Health Professions, ZHAW Zurich University of Applied Sciences, Winterthur, Switzerland

**Keywords:** proprioception, motor control, stochastic resonance, noise-enhanced, vibration-induced, afferent stimulation, sensorimotor, sensory-motor

## Abstract

Stochastic stimulation has been shown to improve movement, balance, the sense of touch, and may also improve position sense. This stimulation can be non-invasive and may be a simple technology to enhance proprioception. In this study, we investigated whether sub-threshold stochastic tactile stimulation of mechanoreceptors reduces age-related errors in wrist position estimation. Fifteen young (24.5±1.5y) and 23 elderly (71.7±7.3y) unimpaired, right-handed adults completed a wrist position gauge-matching experiment. In each trial, the participant's concealed wrist was moved to a target position between 10 and 30° of wrist flexion or extension by a robotic manipulandum. The participant then estimated the wrist's position on a virtual gauge. During half of the trials, sub-threshold stochastic tactile stimulation was applied to the wrist muscle tendon areas. Stochastic stimulation did not significantly influence wrist position sense. In the elderly group, estimation errors decreased non-significantly when stimulation was applied compared to the trials without stimulation [mean constant error reduction $\Delta \mu \left(\theta_{con}^{of}\right)=0.8{}^{\circ}$ in flexion and $\Delta \mu \left(\theta_{con}^{oe}\right)=0.7{}^{\circ}$ in extension direction, *p* = 0.95]. This effect was less pronounced in the young group [$\Delta \mu \left(\theta_{con}^{y}\right)=0.2{}^{\circ}$ in flexion and in extension direction, *p* = 0.99]. These improvements did not yield a relevant effect size (*Cohen's d* < 0.1). Estimation errors increased with target angle magnitude in both movement directions. In young participants, estimation errors were non-symmetric, with estimations in flexion [$\mu \left(\theta_{con}^{yf}\right)=1.8{}^{\circ}$, $\sigma \left(\theta_{con}^{yf}\right)=7.0{}^{\circ}$] being significantly more accurate than in extension [$\mu \left(\theta_{con}^{ye}\right)=8.3{}^{\circ}$, $\sigma \left(\theta_{con}^{ye}\right)=9.3{}^{\circ}$, *p* < 0.01]. This asymmetry was not present in the elderly group, where estimations in flexion [$\mu \left(\theta_{con}^{of}\right)=7.5{}^{\circ}$, $\sigma \left(\theta_{con}^{of}\right)=9.8{}^{\circ}$] were similar to extension [$\mu \left(\theta_{con}^{oe}\right)=7.7{}^{\circ}$, $\sigma \left(\theta_{con}^{oe}\right)=9.3{}^{\circ}$]. Hence, young and elderly participants performed equally in extension direction, whereas wrist position sense in flexion direction deteriorated with age (*p* < 0.01). Though unimpaired elderly adults did not benefit from stochastic stimulation, it cannot be deduced that individuals with more severe impairments of their sensory system do not profit from this treatment. While the errors in estimating wrist position are symmetric in flexion and extension in elderly adults, young adults are more accurate when estimating wrist flexion, an effect that has not been described before.

## 1. Introduction

Proprioception is defined as the sense of movement, position, effort, and balance (Proske and Gandevia, 2018). In the planning and execution of voluntary movement, proprioceptive feedback plays a key role. Humans possess various mechanoreceptors in the muscles, tendons, joints, and skin contributing to proprioception. For example, cutaneous receptors provide information about skin stretch and contribute to the sense of position and movement (Gandevia et al., 1994; Gilman, 2002; Lee et al., 2013; Martin et al., 2015). Muscle spindles, located in parallel to the extrafusal muscle fibers, encode passive and active muscle stretch as well as the rate of stretch (Chen and Poppele, 1973; Granit, 1975; Burke et al., 1976; Roll and Vedel, 1982; Roll et al., 1989). Both position sense and movement sense emerge from muscle spindle ensemble information (Vallbo, 1970; Loeb et al., 1990; Prochazka and Gorassini, 1998; Proske and Gandevia, 2012, 2018).

Proprioceptive deficits are often observed in pathologies such as stroke or Parkinson's disease and accompany loss of motor control and function (Konczak et al., 2009; Findlater et al., 2016; Rinderknecht et al., 2017). When the firing threshold of the muscle spindles rises due to a deterioration of the proprioceptive system, greater stimulation intensities are required to activate the spindles. The frequency/stretch relationship is altered and proprioceptive information distorted. However, muscle spindles can be activated by external tactile stimulation. If a tactile vibratory stimulus is applied at a uniform frequency to the muscles or their tendons, the spindles respond with firings in a 1:1 or sub-harmonic manner (Burke et al., 1976; Roll et al., 1989). When the stimulus is strong enough, an illusion of position or movement can be perceived at the respective joint (Verschueren et al., 2002; Romaiguére et al., 2003; Seizova-Cajic et al., 2007; Roll et al., 2009; Rinderknecht et al., 2013).

Stochastic resonance describes the effect that an unresponsive system becomes responsive to a stimulus when random (stochastic) noise is superimposed (Benzi et al., 1981). To this end, the noise level has to be below the stimulus detection threshold of the system. Accordingly, sub-threshold stochastic noise can be used to increase proprioceptive awareness of individuals affected by degraded proprioception (Cordo et al., 1996; Gopalai et al., 2016): Standing on a sub-threshold vibratory plate has been shown to reduce postural sway in elderly, individuals after stroke, with cerebral palsy, diabetes, or functional ankle instability, indicating improved balance (Collins et al., 2003; Priplata et al., 2006; Ross, 2007; Ross et al., 2013; Zarkou et al., 2018). Moreover, the effect of stochastic noise increased with the severity of deficits: The lower the baseline balance abilities, the higher the impact of the sub-threshold stimulus (Priplata et al., 2004). Similarly, vibrating insoles were shown to reduce compensatory movements during walking in elderly subjects with large gait instability, while they destabilized individuals that had a safe amount of baseline movement variability (Stephen et al., 2014). Besides movement stability and balance, stochastic stimulation also improved the detection of movement onset at the ankle (Ribot-Ciscar et al., 2013).

When applied at the upper extremities, tactile stimulation above tendons reduced compensatory movements during targeted arm movements (Conrad et al., 2015). An optimal level of sub-threshold tactile or electrical noise has also been found to enhance tactile sensation and detection of mechanical stimuli at the finger tips (Collins and Imhoff, 1996; Richardson et al., 1998).

Taken together, these findings support that stochastic stimulation has a positive effect on balance, movement, and the sensation of touch. However, it remains unclear to what extent sub-threshold stochastic tactile stimulation can influence the position sense of the upper extremities.

In this study, we investigated the effect of sub-threshold stochastic tactile stimulation on wrist position sense. While previous studies on stochastic stimulation mostly focused on a motor response of the sensory-motor system, we aimed to examine how proprioception itself is affected when stochastic stimulation is applied. As proprioception deteriorates with age (Adamo et al., 2007; Wright et al., 2011; Rinderknecht et al., 2017), it was hypothesized that elderly adults estimate their wrist's position less accurately than young adults. Furthermore, it was hypothesized that sub-threshold stochastic tactile stimulation applied at the wrist joint reduces the errors made when estimating the wrist's position in elderly adults. The same effect was expected to be minuscule in young adults.

## 2. Methods

### 2.1. Stimulation Characteristics

To influence position sense, the stochastic stimulation employed in this study ought to replicate noise in the frequency band of muscle spindle activity. In the muscle spindle, type Ia (primary or dynamic) afferent fibers convey the rate of change of muscle length and therefore movement. Type Ia fibers respond well to stimulation in the frequency band from 80 to 100Hz (maximum 150Hz) (Roll et al., 1989). Additionally, type II (secondary or static) afferent fibers encode muscle length and therefore joint position. Type II fibers respond well to stimulation in the frequency band from 10 to 30Hz (maximum 60Hz) (Roll et al., 1989). In this study, the frequency band for the stochastic stimulation was set to range from 20 to 120Hz.

In the healthy human body, most physiological patterns such as breathing or heart rates show colored noise characteristics (Sejdié and Lipsitz, 2013), i.e., in those signals, the amplitudes of lower frequency components are typically greater than those of higher frequency components. Colored noise is considered to be superior to white noise for enhancing signal detectability (Duan et al., 2014). In this study, a colored noise spectrum was selected for the stochastic stimulation to predominantly influence type II fibers and therefore joint position sense.

To evoke stochastic resonance effects, the stimulation amplitude should be between 60 and 90% of the participants' stimulus detection threshold (Priplata et al., 2006; Seo et al., 2014; Stephen et al., 2014). In this study, the stimulus detection thresholds were assessed experimentally using the method of ascending and descending limits (Ehrenstein and Ehrenstein, 1999). Before each experimental session, thresholds were assessed for the ventral and dorsal sides of the forearm separately. The stimulation was then set to 80% of each threshold. To avoid an imbalance of agonist-antagonistic spindle signals, which may bias movement or position sense, agonistic and antagonistic joint sides were always stimulated simultaneously (Gilhodes et al., 1986; Roll et al., 1991).

### 2.2. Pneumatic Stimulator

The sub-threshold stochastic tactile stimulation was generated using Soft Pneumatic Actuators (SPAs) (Sonar and Paik, 2016; Sonar et al., 2019). The SPAs consist of two stretchable silicone layers (thickness 0.3 mm, Dragon Skin 30, Smooth-On Inc., Macungie, PA, USA) interleaved with a customizable matrix that creates an inflatable cavity. This allows for the creation of multiple targeted stimulation sites within the small space at the wrist, which would not be possible using commercial tactors. To ensure uniform stimulation conditions around the wrist, the SPAs were covered with an inflatable wrist cuff (OMRON Healthcare Europe B.V., Hoofddorp, The Netherlands).

**Figure 1:**
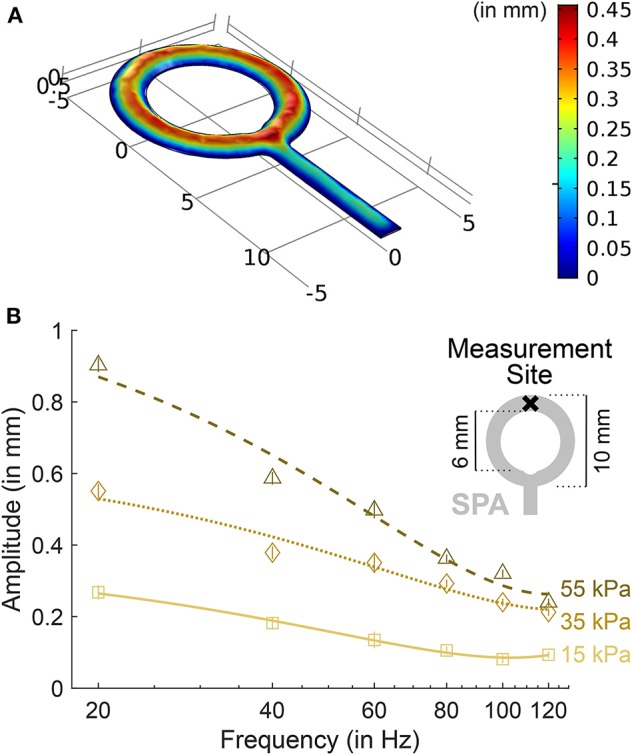
SPA inflation characteristics. **(A)** Dynamic simulation results of SPA inflation show a uniform inflation amplitude throughout the ring structure. Assuming laminar fluid flow for an inlet pressure of 50 kPa, fluid density ρ = 1, 080 kgm^−3^, SPA layer thickness of 0.3mm and Young's modulus *E* = 500 kPa, our current device design showed stable dynamic performance within 0.01 s (corresponding to 100Hz). **(B)** Inflation amplitude as a function of inflation frequency for one exemplary Soft Pneumatic Actuator (SPA). The amplitudes were measured with a laser distance sensor (OWLF 4007 FA S1, Welotec, Laer, Germany) at the top-most point of the SPA. In the plot, markers indicate the amplitude for each frequency averaged over a 1-s measurement. Standard deviations are displayed as vertical bars. Lines indicate 2nd-order polynomial fits. Amplitudes increase proportionally with air pressure. At 120Hz, amplitudes are about one third of the amplitudes at 20Hz.

The design of the SPAs was driven by the stimulation characteristics described in section 2.1 and the wrist's anatomy. Four SPAs were used to stimulate the two wrist flexor (Mm flexores carpi radialis and ulnaris) and three extensor (Mm extensores carpi radialis brevis, radialis longus, and ulnaris) tendons. The SPAs were mounted on the forearm, about four fifths of forearm length from the proximal end. To cover the tendons, one SPA overlaid approximately 100mm^2^. To achieve uniform inflation characteristics across the SPA area, the SPAs were designed as rings (Figure 1A, a more detailed description of the design process can be found in Georgarakis et al., 2017).

The mechanical vibrations of the SPAs were generated using high frequency pneumatic valves (MHE2, Festo AG & Co. KG, Berkheim, Germany). Pressures for extensor SPAs, flexor SPAs and the inflatable cuff were regulated individually (MS4, Festo AG & Co. KG, Berkheim, Germany). The amplitudes of the stochastic stimulation were estimated from static pressure measurements that were obtained during the stimulus detection threshold assessment.

### 2.3. Robotic Manipulandum

The ReFlex is a one degree-of-freedom robotic manipulandum which was used in previous studies to assess wrist and finger proprioception (Chapuis et al., 2010; Rinderknecht et al., 2016). It can move an end-effector to well-controlled positions. In this study, a universal hand interface was used to standardize the initial position of the hand in the robot. In the initial position, the fingers were aligned with the wrist and forearm, while the thumb was slightly abducted. The hand interface was mounted on the motor shaft of the robotic manipulandum DC motor (RE65, Maxon Motor, Sachseln, Switzerland). The motor axis was aligned with the flexion/extension rotation axis of the wrist. The forearm was supported at the elbow and mid-forearm to promote comfort and suppress compensatory movements. The position of the motor was measured with a high-resolution optical encoder (R158, 1 million counts/rev, Gurley Precision Instruments, Troy, NY, USA).

### 2.4. Study Setup

**Figure 2:**
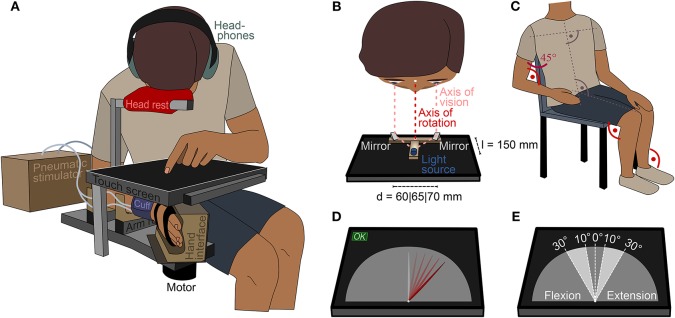
Study setup. **(A)** Participants estimated the position of the right wrist on a touch screen that occluded the wrist. For comfort and to ensure a uniform body position in all trials, participants were asked to use a head rest above the screen. **(B)** The axis of vision was aligned with the axis of rotation of the wrist using a light source and mirror setup. Depending on the eye distance of the participant, the distance of the mirrors was set to *d* = 60mm, *d* = 65mm, or *d* = 70mm. **(C)** Initial position of the participant on the chair before placing the head on the head rest. The elbows, hip, knees, and ankles were flexed at 90°, the shoulder abducted at 45°. **(D)** Screen layout during trials. Except for the needle, the gauge did not show any ticks or indicators. Participants could enter their estimation on the continuous scale by tapping or by dragging the needle and pressing “OK.” The white dot at the bottom of the needle was aligned with the center of rotation of the wrist. **(E)** Presented ranges in wrist flexion and extension direction.

A touch screen was mounted above the robotic manipulandum to visually occlude the wrist (see Figure 2A). Participants used the touch screen to enter their estimation of the position of their wrist with the non-tested hand by moving an indicator on a semi-circular virtual gauge (see Figure 2D).

To minimize errors resulting from visual parallax, the participant's visual axes, the midpoint of the virtual gauge, the axis of rotation of the wrist and the motor axis of the manipulandum must all be aligned. The latter three axes were aligned by design. To aid alignment of the visual axes, two angled mirrors, mounted at eye distance, were set up on the touch screen such that the midpoint between the mirrors was aligned with the midpoint of the virtual gauge (see Figure 2B). A light source was mounted at a distance of *l* = 150mm to the midpoint between two angled mirrors. The light source could only be seen in both mirrors simultaneously if the axes of vision of both eyes were aligned with the midpoint of the mirrors.

The stimulator cuff and the robotic manipulandum were configured to ensure that neither one nor the other would provide additional tactile cues about the lateral wrist position in any state. This was achieved by retracting the cuff and placing the hand in the hand interface such that it was not nudged when the wrist was flexed or extended within the tested range. Participants were seated on a chair next to the robotic manipulandum. The height of the robot was adjusted to the anthropometry of the participant. This ensured that the kinematic chain from the trunk to the shoulder was the same for all participants (see Figure 2C). A uniform body posture is crucial, as even slight changes can influence the whole-body perception of an individual (Roll et al., 1991).

To eliminate auditory cues generated by the robot or stimulator, participants wore over-ear head phones playing white noise. Room lights were dimmed and windows shaded to avoid reflections on the touch screen. The participant and experimenter were separated visually by a black fabric curtain to mitigate visual stimuli.

### 2.5. Participants

Twenty-three (9 female) participants were recruited from an elderly age group (60–88 years, mean 71.7y, standard deviation 7.3y), while 15 (7 female) were recruited from a young adult age group (22–27 years, mean 24.5y, standard deviation 1.5y). To avoid variability due to limb asymmetry effects (Adamo and Martin, 2009), only right-handed participants were admitted, as assessed with the Edinburgh Inventory (Oldfield, 1971). Exclusion criteria included a prior diagnosis of neurological, orthopedic or rheumatologic disease which affects wrist or hand function; any reported self-diagnosed acute or chronic psychiatric disorders; any form of discomfort that affected wrist and finger movement or generated pain during passive wrist and finger movement. The study protocol was approved by the Ethics Commission of ETH Zurich (2016-N-35). All participants were volunteers and provided written informed consent in accordance with the declaration of Helsinki.

### 2.6. Experimental Protocol

**Figure 3:**
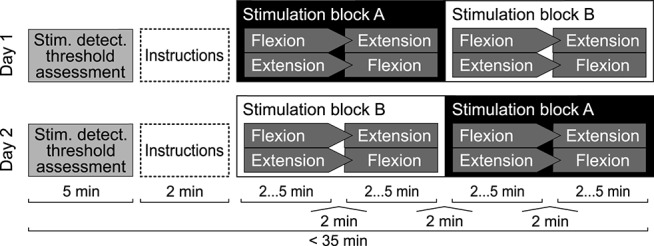
Experimental protocol for a single participant. Either stimulation blocks A or blocks B were presented with stimulation applied, while the other two were presented without stimulation applied. Stimulation levels were set to 80% of the assessed stochastic stimulus detection threshold. Within one stimulation block, one set was presented in wrist flexion and one in wrist extension direction. Orders were randomized using permutations. One set consisted of 21 estimation trials. Per set, each position in the range from 10 to 30° was presented once in random order.

The study was designed as a cross-over trial (see Figure 3). All experiments were conducted over the course of 37 days in Zurich, Switzerland. Participants completed two experimental sessions on two separate days. Prior to the experiment, participants were formally instructed to fully relax their right fingers, wrist and forearm during the experiment, and to focus on the position of their middle finger.

On both days, participants estimated their wrist's position in four sets of 21 trials (Rinderknecht et al., 2013, 2016). Each imposed wrist position represented one unique integer-valued angle in the range from θ_*tar*_ = 10 to 30° from the same initial position at $\theta_{0}=0{}^{\circ}$ (see Figure 2E). For each trial, the ReFlex robot moved the participant's right hand to the target position within one second, following a minimum-jerk trajectory implemented according to (Hogan, 1984):

(1) $$\begin{array}{l} \theta \left(t\right)=\left(10t^{5}-15t^{4}+6t^{5}\right)\theta_{tar} \end{array}$$

Hence, for the tested range [10°…30°], angular velocities reached values in the range [18.75°/*s*…56.25°/*s*], which is well above the movement perception thresholds in the upper extremity (Hall and McCloskey, 1983). We therefore included a two-second delay to wash out movement information, identified heuristically during pilot tests. Thereafter, participants entered the estimation by pointing on the touch screen. Participants were allowed to adjust the estimation. No time restriction was imposed.

Stochastic stimulation was applied during half of the sets. Participants were blinded toward the stimulation condition and the details of the protocol. All conditions and positions were randomized to mitigate order effects. Before and between sets, the stochastic stimulation was paused for 2 min to wash out potential carry-over effects of the stimulation (Ribot-Ciscar et al., 1998).

### 2.7. Data Analysis

The data analysis was performed in R version 3.4.3 (Kite-Eating Tree). The outcome measure of the study was the constant estimation error θ_*con*_ ∈ ℝ, which is the difference between the estimated angle θ_*est*_ ∈ ℝ and the target angle θ_*tar*_ ∈ ℕ:

(2) $$\begin{array}{l} \theta_{con}=\theta_{est}-\theta_{tar} \end{array}$$

By definition, overestimations correspond to a positive value while underestimations correspond to a negative value regardless of the movement direction (flexion or extension). The constant error reflects the variability in the data. To obtain information about the bias in the data, part of the analysis was extended to also include the absolute error

(3) $$\begin{array}{l} \theta_{abs}=|\theta_{con}| \end{array}$$

Due to the complexity of the protocol design, multiple dependencies of the data on different effects were expected. Hence, the complete data set was fitted to a linear mixed model of the form

(4) $$\begin{array}{l} \left(\frac{\theta_{con}}{\theta_{tar}}\right)\sim \text{Treatment}+\text{Day}+\text{Set}+\left(1|\text{Participant}:\text{Day}:\text{Set}\right)+\epsilon \end{array}$$

where *Day* and *Set* denote protocol parameters (1∣*Participant*:*Day*:*Set*) denotes the random effect, and ϵ denotes residual errors. To facilitate hypothesis testing, the target angle was not included as a fixed effect, but constant errors were normalized by target angle.

The fixed effects *Age Group, Movement Direction*, and *Stimulus Setting* including all their interactions were combined to a *Treatment* variable using the R-function interactions():

(5) $$\begin{array}{l} \text{Treatment }<\text{-}\\ \text{ interaction}(\text{Age Group},\text{Movement Direction},\\ \text{ Stimulus Setting}). \end{array}$$

The model fitting was set to optimize using the REML criterion (Hothorn et al., 2017). The significance of the fixed effects in the model was tested using a likelihood ratio test. A detailed description of the linear mixed model and statistical analysis used in this study can be found in Supplementary Material.

Hypotheses were tested for significance with *post-hoc* tests (simultaneous general linear hypothesis testing). The family-wise error rate was controlled using the Bonferroni-Holm method. The significance level was set to α = 0.05.

## 3. Results

### 3.1. Stimulator Inflation Characteristics

Characterization of the system over the used frequency band from 20 to 120Hz revealed the desired colored frequency spectrum (Georgarakis et al., 2017) (see Figure 1B). Average pressure in the SPAs on flexor side was 14.6 kPa (standard deviation: 6.1 kPa) above ambient, on extensor side 13.7 kPa (6.2 kPa) above ambient, as measured with the pressure sensors in static condition (vibrations off). Average pressure in the wrist cuff was 4.0 kPa (2.7 kPa) above ambient as measured with the pressure sensors in static condition. A pressure difference of 10 kPa corresponds to a SPA inflation amplitude of approximately 0.3mm in the static condition.

### 3.2. Data Post-processing

All 38 participants completed the experiment. Due to a data transfer error, one measurement set was missing from the data. In total, 6,363 data points were collected. After removing estimations that exceeded five standard deviations and involuntary zero-estimations, 6,285 data points remained. Residuals of the linear mixed model were approximately normally distributed. Hence, parametric tests were used. Due to the large sample size, a significance test for normality was not performed (Field, 2000).

The analysis of the goodness of fit of the model using the likelihood ratio test identified the *Treatment* variable as the only effect significant at the α-level (*p* < 0.001), while the fixed effects *Day* (*p* = 0.50) and *Set* (*p* = 0.16) were not significant.

### 3.3. Effect of Aging

**Figure 4:**
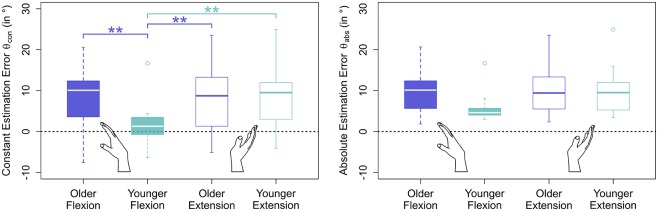
Estimation errors by movement direction and age group. Only blocks without stimulation applied were considered in this plot. While older participants performed symmetrically in flexion and extension direction, young participants performed significantly more accurately in flexion than in extension direction. Moreover, young participants were significantly more accurate in estimating wrist flexion than older in either movement direction, an effect that cannot be seen in extension direction.

**Figure 5:**
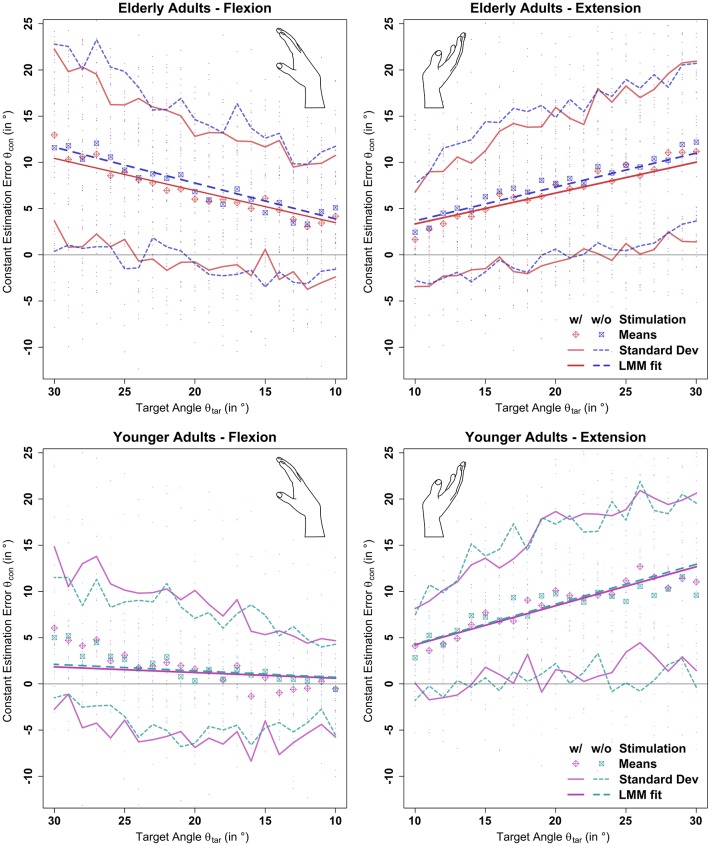
Results of the linear mixed model analysis of the constant estimation error θ_*con*_ with (*w*/) and without (*w*/*o*) stimulation. Stochastic stimulation did not significantly influence wrist position sense. This holds true for extension and flexion direction, though performance in the elderly group was significantly deteriorated in flexion direction. Position estimation errors are shown in the background for each participant. Means and standard deviations were calculated by target angle for the whole population divided by age group, movement direction, and stimulation condition. “LMM fits” show the respective predictions as determined by the linear mixed model fit multiplied by the respective target angle (see section 2.7 and Supplementary Material).

Errors generally increased with target angle size (see **Figure 5** and Table 1). Young participants were significantly more accurate in estimating wrist flexion positions [mean constant error $\mu \left(\theta_{con}^{yf}\right)=1.83{}^{\circ}$, standard deviation $\sigma \left(\theta_{con}^{yf}\right)=7.04{}^{\circ}$] than wrist extension positions [$\mu \left(\theta_{con}^{ye}\right)=8.25{}^{\circ}$, $\sigma \left(\theta_{con}^{ye}\right)=9.33{}^{\circ}$, *p* < 0.01] (see Figure 4). Moreover, young participants were significantly more accurate in estimating wrist flexion positions than elderly in any movement direction (*p* < 0.01). Elderly performed symmetrically in flexion [$\mu \left(\theta_{con}^{of}\right)=7.51{}^{\circ}$, $\sigma \left(\theta_{con}^{of}\right)=9.80{}^{\circ}$] and extension [$\mu \left(\theta_{con}^{oe}\right)=7.68{}^{\circ}$, $\sigma \left(\theta_{con}^{oe}\right)=9.31{}^{\circ}$].

**Table 1:** Constant estimation errors θ_*con*_ = θ_*est*_ − θ_*tar*_ in degrees, averaged over the observed angle space [10°…30°] as well as for the limits of this space.

	**Flexion**						**Extension**					
	**w/ Stimulation**			**w/o Stimulation**			**w/ Stimulation**			**w/o Stimulation**		
	**10°**	**Range**	**30°**	**10°**	**Range**	**30°**	**10°**	**Range**	**30°**	**10°**	**Range**	**30°**
**ELDERLY ADULTS**												
LMM	3.48	6.95	10.43	3.88	7.76	11.64	3.34	6.68	10.02	3.67	7.34	11.00
Mean	4.17	6.96	12.97	5.09	7.51	11.58	1.67	6.96	11.17	2.44	7.68	12.19
SD	7.17	9.14	10.00	7.09	9.80	11.12	5.71	9.41	10.61	6.12	9.31	9.92
**YOUNGER ADULTS**												
LMM	0.61	1.22	1.84	0.70	1.41	2.11	4.23	8.45	12.68	4.31	8.63	12.94
Mean	−0.54	1.69	6.04	−0.62	1.83	5.00	4.11	8.50	11.03	2.83	8.25	9.59
SD	6.14	8.34	10.13	5.55	7.04	7.07	5.56	8.76	10.25	5.41	9.33	10.93

*LMM: Values predicted from the angle normalized linear mixed model, multiplied with the respective target angle to ease interpretation; Mean: Mean value by target angle from original data; SD: Standard deviation by target angle from original data*.

### 3.4. Effect of Stochastic Stimulation

Stochastic stimulation did not significantly alter the constant estimation error θ_*con*_ in the elderly (*p* = 0.95) or young (*p* = 0.99) group. Raw data points, means per target angle, and data fits derived from the linear mixed model analysis per age group, movement direction, and stimulus setting are shown in Figure 5. Detailed information about the constant estimation error θ_*con*_ is summarized in Table 1. The results for the normalized constant estimation error θ_*con*_/θ_*tar*_ can be found in Supplementary Material.

In the elderly group, the average estimation errors in flexion direction made in trials with stimulation were 0.8° lower than in trials without stimulation. In extension direction the average estimation errors made in trials with stimulation were 0.7° lower than in trials without stimulation. In the young group, both in flexion and extension direction the average estimation errors made in trials with stimulation were 0.2° lower than in trials without stimulation. However, none of the stimulation effects yielded a relevant effect size (*Cohen's d* < 0.1).

## 4. Discussion

### 4.1. Young Adults Performed Better in Flexion Direction Than Elderly Adults, However, Both Groups Performed Similarly in Extension Direction

During the majority of daily living tasks, the hand is stabilized in a slightly extended wrist position, employing both flexor and extensor muscles (Salonikidis et al., 2011). Still, wrist position sense is more accurate in flexion direction than in extension direction (Divekar and John, 2013). Accordingly, young adults performed better in estimating flexion positions compared to estimating extension positions. Yet, it remains unclear why the performance of elderly participants was inferior in flexion direction when compared to young participants, but similar in extension direction.

One possible explanation is that performance degrades with age primarily in wrist flexion. Wrist flexor muscles have a larger spindle density than wrist extensor muscles, and are larger in diameter (Banks, 2006; Wright et al., 2011). Thus, the relative degradation of muscle spindles in atrophying wrist flexor muscles may be larger than in atrophying wrist extensor muscles. Proprioception relies on the ensemble information from both agonistic and antagonistic muscles (Gilhodes et al., 1986; Roll et al., 1989). While wrist extension largely relies on wrist extensor muscles, wrist flexion is typically performed in co-activation of flexors and extensors (Salonikidis et al., 2011). The relatively larger deterioration of wrist flexors with age may affect wrist flexion position sense particularly, while it affects wrist extension position sense to a lesser extent.

In addition to muscle spindles, skin stretch has been identified as a source of proprioceptive information (Gandevia et al., 1994; Proske and Gandevia, 2012). As we were applying sub-threshold stochastic tactile stimulation to the tendons through the skin, cutaneous receptors were also stimulated. Different rates of skin slackening or degradation of cutaneous receptors on the ventral and dorsal side of the forearm may also contribute to the discrepancy in performance deterioration between wrist flexion and extension direction.

### 4.2. Initial Position Potentially Lead to Wrist Extensor Pretension

Like in our setup, a large part of the studies on wrist position sense selected an initial position where the metacarpals are aligned with the forearm (Jones et al., 2001; Kamper et al., 2001; Gandevia et al., 2006; Wright et al., 2011; Rinderknecht et al., 2016). This initial position might have led to a pretension in the extensor muscles and therefore have increased the error particularly at larger extension angles. The wrist and fingers are fully relaxed when the wrist is extended at 30° and the fingers are loosely flexed (Gandevia et al., 1994). The initial position selected for this study might therefore lead to increased errors in extension as tension in the flexors is reached only at larger extension angles. However, the observation that only young adults perform better in flexion does not support this theory.

### 4.3. Body Posture Potentially Caused Visual Parallax and Influenced Whole-Body Perception

We developed a new method to align the visual axes of participants with the rotation axis of their wrist. Nevertheless, different head positions in combination with visual parallax may have increased the variability in the data within sets and sessions and between participants. Due to a preferred head position angle and visual parallax, this effect could have caused a bias in one movement direction (flexion or extension), as seen in the young group. However, this bias is not visible in elderly, where performance is similar in flexion and extension. For all participant groups and study conditions, estimation errors became larger proportionally to the target angle magnitude, a tendency that has been discussed before (Rinderknecht et al., 2016).

During the study, the body posture of the participants was clearly defined. This was done to ensure that variability in body posture would not influence the results. The mutual influence of body posture and wrist position has been uncovered before (Roll et al., 1991). The specific body posture configuration in this study might have promoted wrist flexion position estimations, and hampered wrist extension position estimations. Further, the direction of gaze has been shown to influence whole-body perception in experiments where vibratory stimulation of the extra-ocular muscles elicited whole body shifts (Roll et al., 1991). Since the placement of the input gauge forced subjects to look left to estimate wrist flexion positions, and right to estimate wrist extension positions, the whole-body configuration was different during their estimation. As we did not perform a warm up or trials in which participants could see their wrist and hand, we cannot compare their performance with the wrist occluded to a baseline in which their wrist was visible.

### 4.4. Sub-threshold Stochastic Tactile Stimulation Did Not Influence Wrist Position Sense

Though accuracy in wrist position estimation significantly deteriorated with age in flexion direction, stochastic stimulation did not influence estimation accuracy in either movement direction. In elderly adults, estimation errors were 0.8° lower in flexion direction trials with applied stimulation compared to trials without applied stimulation (see Figure 5). This corresponds to an error reduction of 12% in the tested range. For comparison, in studies on noise-enhanced balance control in unimpaired elderly adults, sway is typically reduced by <10% (Collins et al., 2003).

However, when considering effect size measures, stochastic stimulation seems to have a larger effect on sway (*Cohen's d* > 1, calculated from reported data Collins et al., 2003) than on wrist position sense (*Cohen's d* < 0.1) (Coe, 2002). Hence, the lower sensitivity and larger variability in wrist position sense when compared to the sense of balance and sway may have induced the inconclusive results. Nevertheless, for a hand of length 200 mm, an error reduction of 0.8° at the wrist corresponds to an error reduction of 2.8 mm at the finger tips. Though our findings did not reveal a relevant effect size, this difference may be meaningful during activities of daily living that require fine motor skills, such as reaching for small objects.

All participants recruited for this study were adults without upper limb impairments. We assumed that in this cohort, aging would be the primary cause of inferior wrist position sense. However, some elderly participants outperformed even the young group. Physically active, high-functioning elderly adults have shown to perform well in proprioceptive tasks, and might therefore benefit only moderately from stochastic stimulation (Adamo et al., 2009; Dettmer et al., 2016). Individuals with impairments due to diseases and traumatic incidents such as stroke can be affected by larger deteriorations of the proprioceptive system than due to age alone. According to stochastic resonance theory, these individuals are likely to show larger improvements in joint position estimation than the elderly group in this study when stochastic stimulation is applied at their wrist (Seo et al., 2014; Conrad et al., 2015).

The frequency band of the stochastic stimulation applied in this study covered the frequency bands that both type Ia and type II muscle spindle afferent fibers respond to. The stimulation of the movement encoding type Ia afferent fibers might have introduced noise that in turn may have reduced the effect of the stimulation on position sense. Yet, the sub-threshold stochastic stimulation aims to enhance but not to evoke a sensation. It is likely that type Ia fibers were inactive during the position estimation, since the position estimations were entered once a static position was reached, two seconds after the movement had terminated. Hence, it was assumed that any potential disturbance of position sense arising from type Ia fibers is negligible.

Though stochastic stimulation did not significantly influence wrist position sense, it may enhance other modalities in the sensory-motor system. Such modalities could be the sensory-motor integration or a general arousal of the brain. For example, Conrad et al. showed that tactile stimulation applied to the wrist reduces compensatory movements in stroke patients during reaching in unstable workspaces (Conrad et al., 2015). In their study, they applied perceivable, non-stochastic stimulation, set to a constant frequency of 70Hz and a constant amplitude of 1mm, challenging stochastic resonance theory. This stimulation might not have enhanced the spindle reactivity to certain stimuli, but the overall activation of the sensory-motor system.

### 4.5. Gauge Matching Paradigm May Have Reduced Effectiveness of Stochastic Stimulation

Position sense is commonly assessed using one of two paradigms: either a matching paradigm, in which the position of a limb segment is matched with the contra-lateral limb segment; or a pointing paradigm, in which the position of a limb segment is indicated by pointing at the position. While the first requires matching the position of the limb segment relative to the intra-personal space—relying mostly on muscle spindle and cutaneous receptor information—the second requires matching the position with respect to the extra-personal space—relying on vision, touch and hearing (Proske, 2005). Matching the position of a limb segment with the contra-lateral limb introduces uncertainty due to limb asymmetry and dominance (Adamo and Martin, 2009; Adamo et al., 2012). Therefore, for the presented study a gauge matching paradigm was preferred, in which the participants matched the position of their wrist pointing at a gauge with their contra-lateral hand. The reliability of this approach has been shown before (Rinderknecht et al., 2016). As visual and auditory cues were reduced as much as possible, participants had to rely on their proprioceptors, primarily the muscle spindles and cutaneous receptors, to estimate their wrist position. However, the matching paradigm might have reduced the effectiveness of the stochastic stimulation. It has been shown before that illusory movements evoked by tendon vibration are less pronounced when a pointing paradigm is applied compared to when a matching paradigm is applied (Proske, 2015).

### 4.6. Stimulation Amplitudes Are Consistent With Previous Findings

In previous studies applying sub-threshold tactile stimulation, the resulting stimulation amplitudes were usually not reported (Priplata et al., 2002; Collins et al., 2003; Seo et al., 2014, 2015; Stephen et al., 2014; Dettmer et al., 2016). However, this information is an important requirement for future study designs. Here, the amplitudes of the stochastic stimulation were estimated from the static pressure measurements that were obtained before the first block. The amplitudes generated in vibrating mode are lower than the static amplitudes due to visco-dynamic effects, and can be estimated to be below 0.3mm. This is in correspondence with previous findings that tested stochastic stimulation with amplitudes between 0.0mm and 0.3mm and found a peak in performance enhancement for stimulation amplitudes just below 0.1mm (Ribot-Ciscar et al., 2013).

To gain a better understanding of the mechanisms involved when stochastic stimulation is applied to different body sites, further studies using kinematic outcome measures, EEG or MRI imaging are required, especially when the upper extremities are concerned. In contrast to electromagnetic tactors frequently used in previous studies (Priplata et al., 2006; Ribot-Ciscar et al., 2013; Ross et al., 2013; Seo et al., 2014, 2015), the pneumatic stimulator developed for this study is compatible with MRI systems and therefore ideal for such future experiments.

## 5. Conclusion

While the performance of wrist position estimation was symmetric in elderly adults, young adults were significantly better at estimating wrist flexion positions. To the best of our knowledge, this effect has not been described before. Regardless of the movement direction, sub-threshold stochastic tactile stimulation did not significantly influence wrist position estimation accuracy in highly functioning elderly adults. As expected, the same was true for young adults. However, as elderly adults showed a tendency toward increased position estimation accuracy when stochastic stimulation was applied, we cannot exclude that individuals with impaired wrist proprioception benefit from stochastic stimulation.

## Data Availability Statement

The raw data supporting the conclusions of this article will be made available by the authors, without undue reservation, to any qualified researcher.

## Ethics Statement

The studies involving human participants were reviewed and approved by ETH Ethics Commission. The participants provided their written informed consent to participate in this study.

## Author Contributions

A-MG, JD, and HS designed and constructed the pneumatic stimulator. WP configured the robotic manipulandum. A-MG, VK-M, JD, BM, MR, and OL designed the study protocol. A-MG and WP collected the data. A-MG performed the statistical analysis and prepared the manuscript. All authors were involved in the interpretation of results, provided critical feedback on the manuscript, and read and approved the final version.

### Conflict of Interest

The authors declare that the research was conducted in the absence of any commercial or financial relationships that could be construed as a potential conflict of interest.
