# Supplementary material for: Age-Dependent Asymmetry of Wrist Position Sense Is Not Influenced by Stochastic Tactile Stimulation
Source: Front Hum Neurosci. 2020 Mar 3;14:65. doi: 10.3389/fnhum.2020.00065 (PMC7063068; doi:10.3389/fnhum.2020.00065)
Supplement: Supplementary file 1 [file Data_Sheet_1.pdf]

Statistical Analysis

1 LINEAR MIXED MODEL

The data analysis was performed in R version 3.4.3 (Kite-Eating Tree, packages: lme4 Bates et al. (2015, 2007), lmerTest Kuznetsova et al. (2016), multcomp Hothorn et al. (2017) and car Fox et al. (2012)). The complete data set was fitted to a linear mixed model of the form

$$\theta \sim \sum_i X_i + \sum_j Y_j + \epsilon,$$

where θ is the dependent variable, X_i denote fixed effects, Y_j denote random effects, and ϵ denotes residual errors.

The data was fitted to the linear mixed model using the R-function `lmer()`, which was set to optimize using the REML criterion:

```
LMM <- lmer(ConstErr/TargetAngle ~  Treatment +
                                     Day +
                                     Set +
                                     (1 | Participant:Day:Set) )
```

where `Treatment` is a super-factor combining the fixed effects `StimulusSetting`, `AgeGroup` and `MovementDirection` and all their interactions.

```
Treatment <- interaction(StimulusSetting, AgeGroup, MovementDirection)
```

2 DEPENDENT VARIABLE

ConstErr$|_{\text{cont.}}$. This continuous dependent variable denotes the constant estimation error, i.e. the error including direction participants made when estimating the position of their wrist.

TargetAngle$|_{\text{cont.}}$. This continuous dependent variable denotes the target angle, i.e. the angle that was presented to the participant.

3 FIXED EFFECTS

The following fixed effects X_i were used to reflect the dependencies of the data due to the study protocol. The number of levels of an effect are indicated at the bottom of the vertical bars.

AgeGroup$|_2$. This fixed effect denotes the age group the participant belonged to, either younger or older.

StimulusSetting$|_2$. This fixed effect denotes whether or not stimulation was applied for the given data point.

MovementDirection$|_2$. This fixed effect denotes the direction of the presented position, either in wrist flexion or wrist extension direction.

Day$|_2$. This fixed effect denotes whether the data was collected during the first or the second study day.

Set$|_4$. This fixed effect denotes the order of the sets within one study session.

The fixed effects StimulusSetting, AgeGroup and MovementDirection were combined to a “Treatment” effect. This greatly facilitated post-hoc tests and interpretation of results.

The significance of fixed effects was tested using the `drop1(···, test="Chisq")`-function which applies a likelihood ratio test comparing the models with and without the respective fixed effect.

4 RANDOM EFFECTS

The following random effect Y_i was added to allow for variability in the data due to the study design:

(1 | Participant:Set:Day). This random effect allows for variability in the intercepts for each participant per study day and set.

5 HYPOTHESIS TESTING

According to the study design and after data inspection, the following hypotheses were tested:

H1 Stochastic stimulation improves wrist position sense in elderly adults.
H2 Stochastic stimulation improves wrist position sense in younger adults.
H3 Younger adults estimate their wrist position in flexion direction more accurately than in extension direction.
H4 Younger adults estimate their wrist position in flexion direction more accurately than elderly adults.
H5 Younger adults estimate their wrist position in flexion direction more accurately than elderly adults in extension direction.

Hypotheses were tested for significance with post-hoc tests using the R-function `glht()` for simultaneous general linear hypothesis testing:

```
glhtTest <- glht(lmmFin,linfct = K)
summary(glhtTest)
```

where K is a 5×12 contrast matrix in which the rows represent the five hypotheses to be tested, and the columns represent the fixed effects and their interactions.

Table S1. Contrast Matrix K. Notation for fixed effects: “S” for Stimulation on, “N” for stimulation off, “O” for elderly, “Y” for younger,“F” for flexion, “E” for extension, “:” denote interactions. “D2” for day, “S2, S3, S4” for sets.

	Intercept	S:O:E	N:Y:E	S:Y:E	N:O:F	S:O:F	N:Y:F	S:Y:F	D2	S2	S3	S4
H1	0	1	0	0	-1	1	0	0	0	0	0	0
H2	0	0	-1	1	0	0	-1	1	0	0	0	0
H3	0	0	0	0	-1	0	1	0	0	0	0	0
H4	0	0	0	0	0	0	1	0	0	0	0	0
H5	0	0	-1	0	0	0	1	0	0	0	0	0

To test the hypotheses, the respective fixed effects were set in contrast. The function `glht()` controls the family-wise error rate by correcting for multiple testing using the Bonferroni-Holm method. The significance level was set at $\alpha = 0.05$.

6 SUPPLEMENTARY RESULTS

Table S2 and Figure S1 show the raw data results and the results of the linear mixed model analysis for the constant error normalized by target angle $\theta_{con}/\theta_{tar}$.

Table S2. Results of the linear mixed model analysis of the constant estimation error normalized by target angle $\theta_{con}/\theta_{tar}$ with $(w/)$ and without (w/o) stimulation, averaged over the observed angle space $[10° \ldots 30°]$ as well as for the limits of this space.

	**Flexion**						**Extension**					
	w/ Stimulation			w/o Stimulation			w/ Stimulation			w/o Stimulation		
	10°	Range	30°	10°	Range	30°	10°	Range	30°	10°	Range	30°
**ELDERLY ADULTS**												
LMM	0.35	0.35	0.35	0.39	0.39	0.39	0.33	0.33	0.33	0.37	0.37	0.37
Mean	0.42	0.35	0.43	0.51	0.38	0.39	0.17	0.35	0.37	0.24	0.38	0.41
SD	0.72	0.46	0.33	0.71	0.49	0.37	0.57	0.47	0.35	0.61	0.47	0.33
**YOUNGER ADULTS**												
LMM	0.06	0.06	0.06	0.07	0.07	0.07	0.42	0.42	0.42	0.43	0.43	0.43
Mean	-0.05	0.08	0.2	-0.06	0.09	0.17	0.41	0.43	0.37	0.28	0.41	0.32
SD	0.61	0.42	0.34	0.55	0.35	0.24	0.56	0.44	0.34	0.54	0.47	0.36

LMM: Values predicted from the angle normalized linear mixed model; Mean: Mean value by target angle from original data; SD: Standard deviation by target angle from original data.

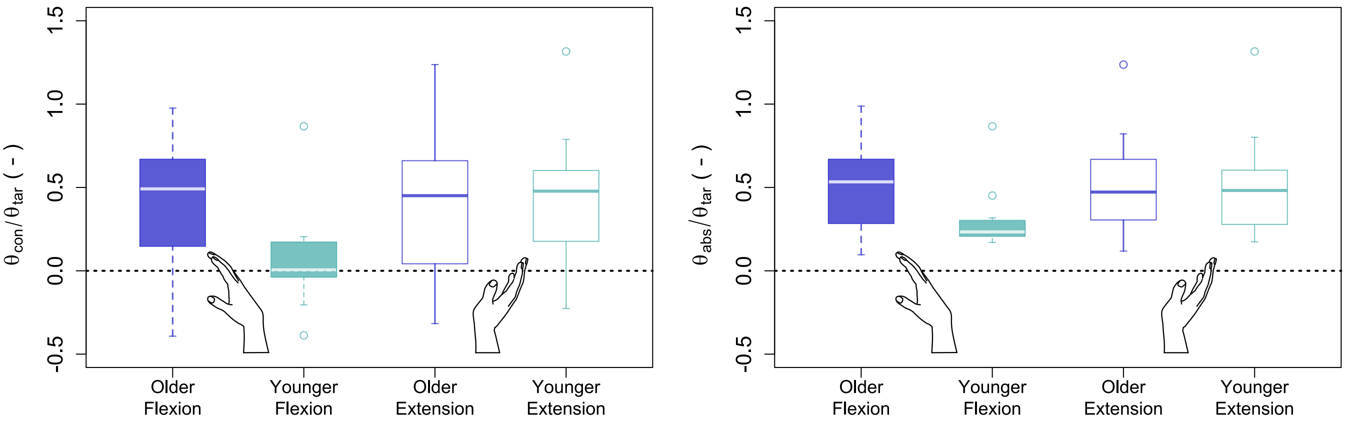

Figure S1. Estimation errors normalized by target angle by movement direction and age group. Only blocks without stimulation applied were considered in this plot. While older participants performed symmetrically in flexion and extension direction, young participants performed significantly more accurate in flexion than in extension direction. Moreover, young participants were significantly more accurate in estimating wrist flexion than older in either movement direction, an effect that cannot be seen in extension direction.

REFERENCES

Bates, D., Mächler, M., Bolker, B., and Walker, S. (2015). Fitting linear mixed-effects models using lme4. *Journal of Statistical Software* 67, 1–48. doi:10.18637/jss.v067.i01

Bates, D., Sarkar, D., Bates, M. D., and Matrix, L. (2007). *The lme4 package*. R package version

Fox, J., Weisberg, S., Adler, D., Bates, D., Baud-Bovy, G., Ellison, S., et al. (2012). *Package 'car'*. R package version

Hothorn, T., Bretz, F., Westfall, P., Heiberger, R. M., Schuetzenmeister, A., and Scheibe, S. (2017). *Package 'multcomp'*. R package version

Kuznetsova, A., Bruun Brockhoff, P., and Haubo Bojesen Christensen, R. (2016). *lmerTest: Tests in Linear Mixed Effects Models*. R package version 2.0-32
